# Plasma proteomic characterization of colorectal cancer patients with FOLFOX chemotherapy by integrated proteomics technology

**DOI:** 10.1186/s12014-024-09454-z

**Published:** 2024-04-05

**Authors:** Xi Wang, Keren Zhang, Wan He, Luobin Zhang, Biwei Gao, Ruijun Tian, Ruilian Xu

**Affiliations:** 1 The Second Clinical Medical College of Jinan University, the First Affiliated Hospital of Southern University of Science and Technology, Shenzhen People’s Hospital, Shenzhen, 518020 China; 2 The First Affiliated Hospital, Jinan University, Guangzhou, 510632 China; 3 Department of Chemistry and Research Center for Chemical Biology and Omics Analysis, School of Science, Southern University of Science and Technology, Shenzhen, 518055 China

**Keywords:** Colorectal cancer, Plasma proteome, MS-based proteomics, Machine learning, PRM validation

## Abstract

**Background:**

Colorectal Cancer (CRC) is a prevalent form of cancer, and the effectiveness of the main postoperative chemotherapy treatment, FOLFOX, varies among patients. In this study, we aimed to identify potential biomarkers for predicting the prognosis of CRC patients treated with FOLFOX through plasma proteomic characterization.

**Methods:**

Using a fully integrated sample preparation technology SISPROT-based proteomics workflow, we achieved deep proteome coverage and trained a machine learning model from a discovery cohort of 90 CRC patients to differentiate FOLFOX-sensitive and FOLFOX-resistant patients. The model was then validated by targeted proteomics on an independent test cohort of 26 patients.

**Results:**

We achieved deep proteome coverage of 831 protein groups in total and 536 protein groups in average for non-depleted plasma from CRC patients by using a Orbitrap Exploris 240 with moderate sensitivity. Our results revealed distinct molecular changes in FOLFOX-sensitive and FOLFOX-resistant patients. We confidently identified known prognostic biomarkers for colorectal cancer, such as S100A4, LGALS1, and FABP5. The classifier based on the biomarker panel demonstrated a promised AUC value of 0.908 with 93% accuracy. Additionally, we established a protein panel to predict FOLFOX effectiveness, and several proteins within the panel were validated using targeted proteomic methods.

**Conclusions:**

Our study sheds light on the pathways affected in CRC patients treated with FOLFOX chemotherapy and identifies potential biomarkers that could be valuable for prognosis prediction. Our findings showed the potential of mass spectrometry-based proteomics and machine learning as an unbiased and systematic approach for discovering biomarkers in CRC.

**Supplementary Information:**

The online version contains supplementary material available at 10.1186/s12014-024-09454-z.

## Introduction

Colorectal cancer (CRC) continues to be a significant global health burden, ranking as the third most prevalent cancer type even in 2022 [1]. Postoperative chemotherapy for Stage II/III CRC patients typically involves the FOLFOX regimen, which is a chemotherapy combination of folinic acid, 5-fluorouracil and oxaliplatin [2]. Despite its standard use, only a small percentage of stage II and III CRC patients achieve successful outcomes [3]. The lack of specific guidelines to identify patients who will benefit from FOLFOX treatment leads to a large proportion of CRC patients undergoing potentially unnecessary chemotherapy. Therefore, the development of a reliable method for predicting the efficacy of FOLFOX perioperative chemotherapy in colorectal cancer becomes paramount. Accurate prognostic biomarkers play a vital role in cancer diagnosis, with examples such as carbohydrate antigen 125 (CA125) and carcinoembryonic antigen (CEA) linked to poor prognosis and an increased risk of cancer metastasis [4, 5]. Carbohydrate antigen 19–9 (CA19-9) has also been considered for predicting postoperative prognosis in stage III colon cancer [6]. However, current detection methods such as preoperative imaging, tumor grading, and mutation burden have proven inadequate in predicting the response to FOLFOX chemotherapy effectively. To address this challenge, researchers have explored complementary assays, including lymphocyte counts [7] and neutropenia [8], but these may only provide averaged measurements or lack the sensitivity and specificity needed for the complex biological changes in FOLFOX-treated patients. Thus, there is a pressing need for more sensitive and specific diagnostic approaches to guide personalized treatment plans and optimize cancer therapy for CRC patients receiving FOLFOX treatment.

The plasma proteome of CRC patients with distinct responses to FOLFOX treatment is likely to be different due to the shedding of circulating tumor fragments in the blood [9]. Among the promising non-invasive plasma-based approaches, measuring circulating tumor DNA and circulating tumor cells (CTCs) has shown potential in predicting the response to adjuvant chemotherapy in stage II CRC patients [8, 10]. However, both methods suffer from low detection rates and the requirement for relatively large blood samples. Therefore, a more accurate prediction approach requiring less blood volume is urgently needed. Mass spectrometry-based shotgun plasma proteomics has emerged as a systematic approach enabling the identification of dynamic protein changes in the progression of diseases on a proteome scale [11, 12]. Persistent innovations in sample preparation, instrumentation, and data analysis have contributed to CRC plasma biomarker discovery [13, 14]. Notably, Niu et al. utilized the data-independent acquisition (DIA) method to quantify plasma proteins, exploring their diagnostic and prognostic potential [15]. Our group has developed the simple and integrated spintip-based technology (SISPROT) for proteomics sample preparation [16]. Our recent SISPROT workflow allows the streamlined quantification of several hundreds of proteins from a small volume of plasma within 3 h in a multiplex manner [17]. To further enhance plasma proteome coverage, we extended the pipeline with the DIA strategy termed SISPROT-DIA [18]. DIA was adopted for its robustness against contamination, offering promise for clinical proteome studies [19]. Additionally, machine learning has shown potential for biomarker discovery and risk stratification of patients [20, 21].

In this study, we employed the SISPROT-DIA workflow for deep proteome profiling of plasma samples, combining it with machine learning and targeted parallel reaction monitoring (PRM) validation. Our goal is to explore the possibility of using plasma protein panels to predict the outcome of FOLFOX treatment in stage II/III CRC patients. The findings from this study hold the potential to provide valuable insights into personalized treatment strategies for CRC patients receiving FOLFOX chemotherapy.

## Methods

### Human plasma sample collection

Blood samples were prospectively collected from CRC patients undergoing FOLFOX treatment at The People's Hospital of Shenzhen. Ethical approval was obtained from the relevant institutional review board, and written informed consent was obtained from all participants. To prevent coagulation, venous blood was drawn using (Ethylenediaminetetraacetic Acid) EDTA collecting vessels. Subsequently, the blood samples were centrifuged at 4 °C and 1500 ×*g* for 30 min to separate the plasma from cellular components. The extracted plasma samples were promptly stored at − 80 °C to maintain sample integrity until further analysis. Throughout the research process, strict adherence to ethical guidelines and data protection measures ensured patient privacy and confidentiality.

### Proteome sample preparation

For plasma proteome profiling, 1 μL of each plasma sample was diluted and prepared using the SISPROT kit (BayOmics, China) following the manufacturer’s protocol [16]. The SISPROT tip was activated and equilibrated, and then the plasma samples were loaded onto the ion-exchange layer of the SISPROT tip. Afterward, the samples underwent the processes of reduction, alkylation, digestion, and desalting. The resulting peptides were reconstituted in 0.1% formic acid in water mixed with 0.1 × indexed retention time (iRT) reagent (Biognosys, Switzerland), where the iRT peptides served as internal standards to accurately align retention times. The prepared plasma samples were then injected into the LC–MS/MS system, enabling high-precision and sensitive analysis of the plasma proteome.

### Liquid chromatography-tandem mass spectrometry (LC–MS/MS) data acquisition

LC–MS/MS data acquisition was performed using a data-independent acquisition (DIA) method on an Orbitrap Exploris 240 mass spectrometer, coupled with an Ultimate 3000 liquid chromatography system (ThermoFisher, USA). Peptide samples were separated using a self-packed analytical column (100 μm × 20 cm, 1.9 μm C18) at a flow rate of 500 nL/min. The mobile phase A and mobile phase B were 0.1% formic acid in water and in acetonitrile, respectively. A 65-min gradient was employed as follows: 0–2 min, 4–10% B; 2–52 min, 10–28% B; 52–62 min, 28–45% B; 62–64 min, 45–99% B, and the final 1 min held at 99% B.

The mass spectrometry (MS) instrument settings were as follows: the mass range of MS1 was 400–1200 m/z, operating at a resolution of 6000. The automatic gain control (AGC) was set to 300%, and the auto maximum injection time mode was enabled. For the DIA setting, the mass range of 400–1200 m/z was divided into 32 continuous windows for MS2 scans, each acquired at a resolution of 30,000. The maximum injection time for MS2 scans was set to 54 ms, with an AGC target of 1E6. The Stepped normalized collision energy for MS2 scans was distributed to 28, 32, and 36, respectively.

### Data analysis and statistics

Raw data obtained from the LC–MS/MS analysis were processed using Spectronaut software (v16.0, Biognosys, Switzerland) with the library-free directDIA mode. The MS raw files were searched against the reviewed human UniProt FASTA database (20,601entries) with Biognosys (BGS) factory settings. Proteins exhibited more than a 30% missing value rate across all samples were removed. The missing values was imputed with the median value of the respective samples. Following this, we normalized the data using the median protein intensities across each sample to correct technical variations. To ensure reliable comparisons, the original expression intensities of proteins were normalized using the median value of each sample. Statistical analysis involved the use of MetaboAnalyst web server (https://www.metaboanalyst.ca/home.xhtml), which encompassed the imputation of missing values and subsequent data normalization. PLS-DA and heatmap clustering for pattern recognition, and differential analysis with volcano plot analysis to identify statistically significant protein alterations. With the significant changed features, we carried out pathway analysis employing Metascape analysis and the KEGG enrichment by R packages org.Hs.eg.db and clusterProfiler. To identify essential plasma proteome features and establish a robust protein panel for predicting treatment efficacy, we employed a machine learning method called Random Forest. The model establishment and evaluation were carried out using the R packages caret, lattice and randomForest. The R packages pROC [22] were utilized for model establishment and evaluation. To assess the predictive power of the model, an outer resampling method with cross-validation (threefold cross-validation repeated 5 times) was implemented, with receiver operating characteristic (ROC) analysis used for performance evaluation. Data were visualized by R package ggplot2.

### Biomarker combination validation by PRM quantification

Validation of the identified biomarker combination was performed on a new cohort of plasma samples collected and prepared using the same methodology as described for the discovery cohort. The validation data were acquired using parallel reaction monitoring (PRM), a targeted quantification method employing Orbitrap MS technology. The Orbitrap Exploris 240 mass spectrometer with the same LC settings as the previous proteome profiling study was used. During PRM MS data acquisition, the full scan resolution was set at 120,000 with a scan range of 400–1200 m/z and a maximum injection time of 100 ms. For targeted MS2, an isolation window of m/z 0.7 was applied at a resolution of 45,000, coupled with a maximum injection time of 150 ms and an AGC target of 1E5. Subsequent data analysis, including peak filtration and area calculation, was conducted using Skyline software (v21.2).

## Results

### Study design and quality evaluation of the plasma proteome analysis

**Fig. 1:**
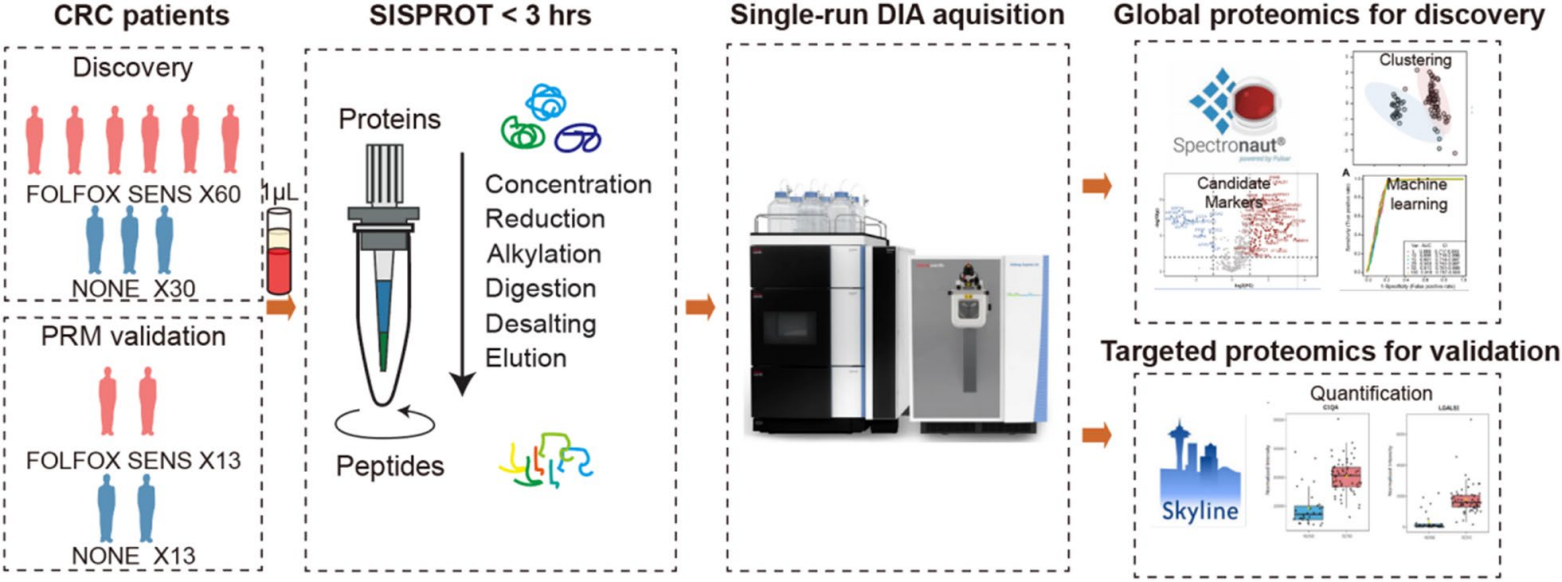
The cohort design and the SISPROT-DIA workflow

In this study, we present a streamlined workflow to investigate the impact of FOLFOX treatment on the plasma proteome of colorectal cancer (CRC) patients. In the discovery phase, we collected plasma samples from 90 CRC patients (Fig. 1). These patients were categorized into two groups, the sensitive group (SENS) consisting of 60 individuals who showed stable recovery and no relapse after surgery and the no-impact group (NONE) comprising 30 patients whose tumors metastasized. Table 1 provides the basic clinical characteristics of the patients, including age at diagnosis, gender, height, and weight for calculating the body mass index (BMI). We employed appropriate statistical methods to analyze normally distributed values. Additionally, we collected the information of traditional clinical tumor markers, namely CEA, CA19-9, and CA125, which are commonly associated with colorectal cancer (Table 1). The CA125 levels in all patients ranged from 1.6 to 89.68, with a median value of 13.54. NONE group exhibited a higher concentration range of CA125, CEA and CA19-9 as expected, while no significant difference was observed between the two groups (Table 1). This suggests the need for complementary markers to increase prediction accuracy.

**Table 1:** Baseline characteristics of CRC-FOLFOX plasma proteome profiling cohort

Characteristics	Total	SENS	NONE	P-value
Number of samples (n)	90	60	30	–
Age (years, mean ± SD)	57 ± 22	56 ± 20	56 ± 21	0.14
Gender (male, %)	57(63%)	38(63%)	19(63%)	–
BMI (kg/m^2^, mean ± SD)	23.34 ± 2.48	23.34 ± 2.5	23.62 ± 1.5	0.300
CA125 (U/mL)	13.54 (10.01–17.92)	13.10 (10.08–17.84)	13.72 (9.77–21.36)	0.494
CEA (ng/mL)	3.11 (2.14–5.09)	2.61 (1.88–3.93)	3.74 (2.80–16.45)	0.499
CA19-9 (U/mL)	11.19 (7.76–17.81)	10.62 (7.68–13.74)	12.83 (7.62–26.15)	0.500

We employed MS-based proteomics for plasma proteome profiling and subsequent screening of diagnostic markers (Fig. 1). To achieve this, we processed the plasma samples from the discovery cohort using a highly reproducible 3-h proteomics sample preparation method known as SISPROT [16]. The resulting MS-ready peptides were then subjected to LC–MS analysis using data-independent acquisition mode, allowing us to achieve deep quantitative plasma proteome profiling (Fig. 1). The expression matrix of the proteome obtained from the LC–MS analysis was further subjected to functional analysis and utilized to train machine learning models. This approach enabled us to generate protein panels that could be used for prognostic prediction, potentially identifying key markers associated with treatment response and patient outcomes. To validate the potential biomarkers identified through the discovery phase, we utilized the parallel reaction monitoring (PRM) method of targeted proteomics. This validation step allowed us to confirm the presence and abundance of specific proteins of interest, providing additional evidence for the reliability and relevance of our findings (Fig. 1).

**Fig. 2:**
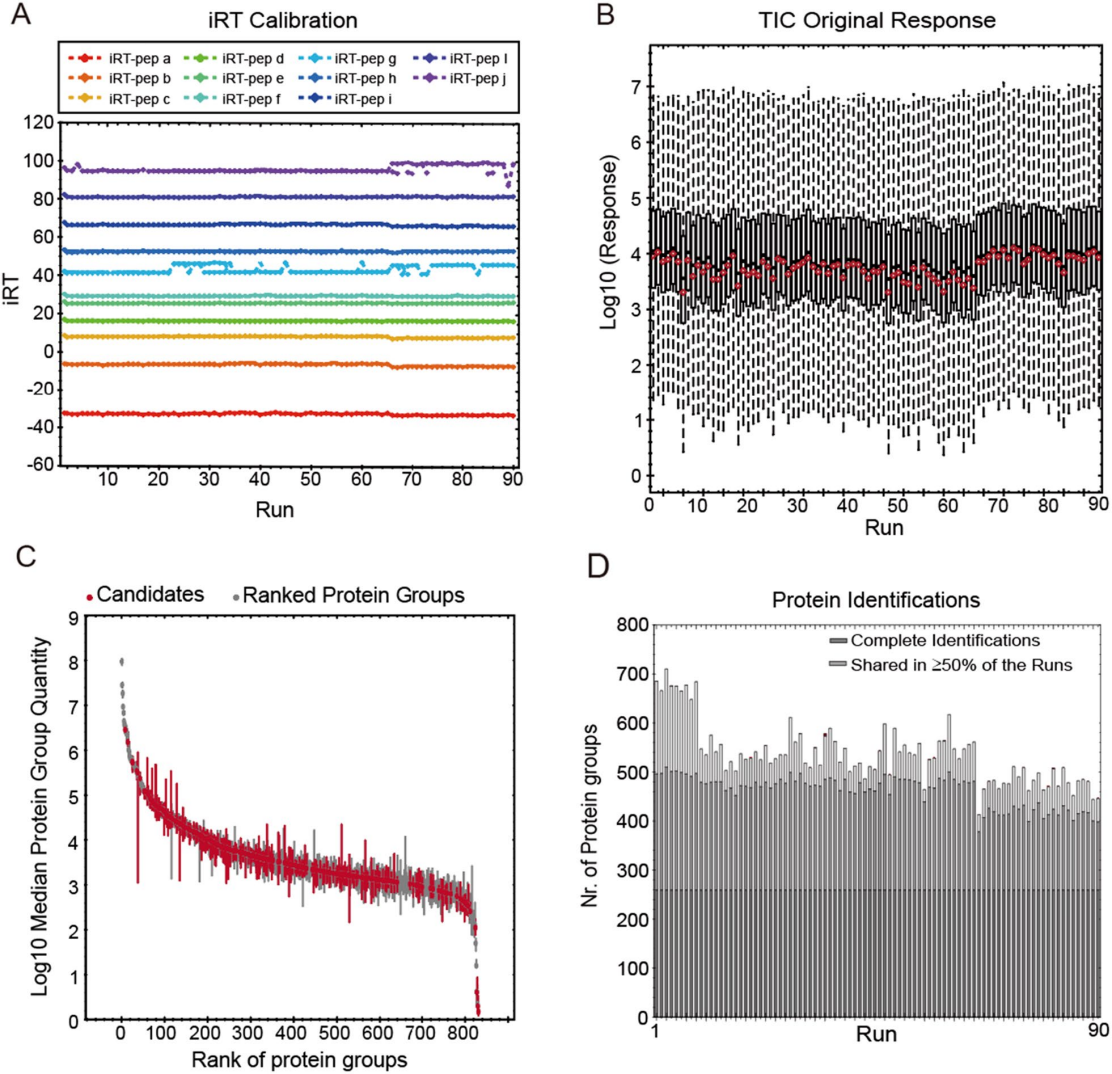
Technical aspect of plasma proteomic profiling of CRC patients. **A** Internal standard retention time LC stability. Each color represents a peptide with its retention time index number. **B** Original response boxplots of MS intensities across all 90 samples. The Red line dots represent the mean value of each sample. **C** Dynamic range of plasma proteome profiling. **D** Protein identification across all 90 samples

To ensure reliable biomarker screening, rigorous quality controls were implemented for LC–MS/MS detection over an extended data acquisition period. Indexed retention time (iRT) perturbation demonstrated the stability of our LC system, with minimal deviations observed at adjusted retention times 40 and 100 (Fig. 2A). Consistent results were confirmed through manual peak comparison, total ion chromatogram, and base peak overlay analyses. MS analysis exhibited related consistency in the original response of total intensity in each group, subsequently normalized by the median (Fig. 2B). Our single-run shotgun proteomic workflow identified 831 protein groups in total from 1 μL plasma samples of 90 CRC patients, covering a broad dynamic range of 8 orders of magnitude (Fig. 2C). After data filtering and normalization, 536 protein groups were quantified on average per sample, showcasing the high quality of our data set (Fig. 2D).

### Plasma proteome profiling of CRC patients undergoing FOLFOX chemotherapy

**Fig. 3:**
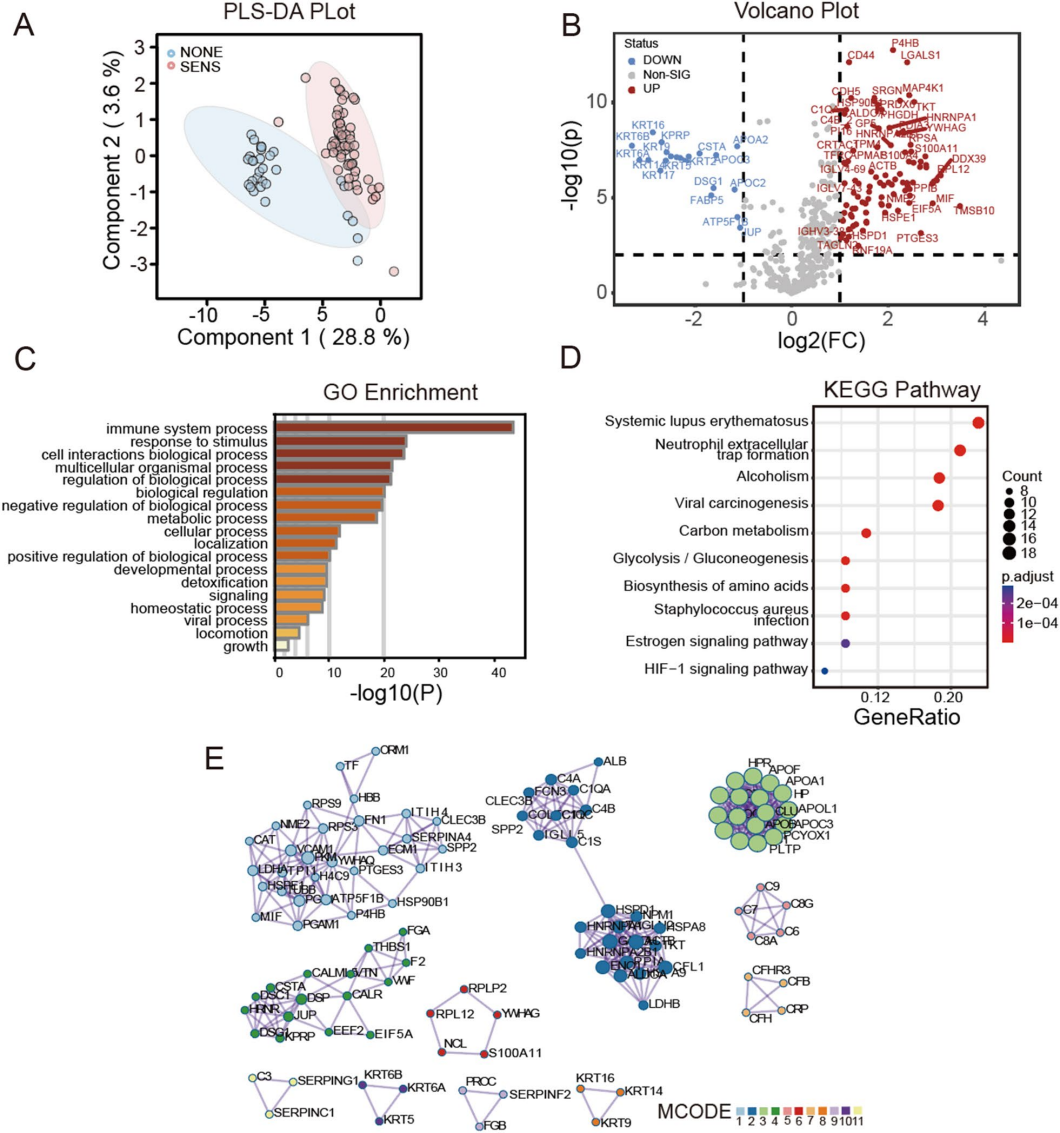
Characterization of the plasma proteome of CRC patients with FOLFOX chemotherapy. **A** Scores plot for Partial least square-discriminant analysis (PLS-DA). The scores plot showed separation between the two groups based on their expression. SENS group showed in red and NONE group in blue circles. **B** Volcano plot representing the difference in plasma expression levels of 115 proteins between the two groups. Red and blue dots indicate proteins with increased and decreased expression levels, respectively. p < 0.01, fold change [SENS/NONE] > 2 (log2FC > 1). **C** Bar chart showing significant canonical pathways (**B**–**H** p-value < 0.05) enriched by Gene Ontology Biological Processes (GOBP). **D** Dots plot showing the Kyoto Encyclopedia of Genes and Genomes (KEGG) enrichment. **E** Molecular Complex Detection (MCODE) networks. Each color represents a cluster of protein–protein interaction

The aim of this study was to investigate the impact of FOLFOX chemotherapy on the plasma proteome of colorectal cancer (CRC) patients and identify potential biomarkers associated with treatment response and patient outcomes. We utilized MS-based proteomics to comprehensively analyze protein expression profiles in CRC patients undergoing FOLFOX treatment. Partial least square-discriminant analysis (PLS-DA), a supervised clustering method, demonstrated a clear separation between the sensitive (SENS) and no-impact (NONE) groups based on their plasma protein expression profiles (Fig. 3A), consistent with sample types. The majority of the variance (PC1) accounted for 28.8% of the data, indicating strong discriminatory power. Volcano plots revealed 257 significant proteins with FDR-corrected p-values < 0.01 and 115 dysregulated proteins with at least a twofold change (log2FC = 1) (Fig. 3B). Among these, 95 proteins were up-regulated, and 20 were down-regulated in the comparison between the SENS and NONE groups. The list of these proteins can be found in Additional file 3: Table S2.

Gene ontology (GO) enrichment analysis on the 257 significant proteins highlighted the immune system process (Fig. 3C), as the most significantly enriched biological process, including complement-related proteins such as complement component C1r (C1R), complement factor D (CFD), complement factor B (CFB), complement factor I (CFI), complement C1s subcomponent (C1S), and complement component C6 (C6). Additionally, the response to stimulus, involving proteins known to be involved in cancer development such as immunoglobulin heavy variable chain, serotransferrin, and CD44 antigen [23], was the second most enriched process. Notably, a significant enrichment in metabolic processes was observed, particularly pyruvate metabolic processes, which have been associated with CRC initiation and cancer progression. Additionally, several enzymes were identified in this category. Notably, a significant enrichment in metabolic processes was observed when inspecting the child’s terms of the gene ontology biological process (GOBP). For instance, proteins involved in pyruvate metabolic processes, including fructose-bisphosphate aldolase A (ALDOA), alpha-enolase (ENO1), glyceraldehyde-3-phosphate dehydrogenase (GAPDH), l-lactate dehydrogenase A (LDHA), l-lactate dehydrogenase B (LDHB), phosphoglycerate mutase 1 (PGAM1), phosphoglycerate kinase 1 (PGK1), pyruvate kinase PKM (PKM), and triosephosphate isomerase (TPI1), were found to be dysregulated. This metabolic process, also known as glycolytic process [24], has been reported to have a strong relationship with CRC initiation and cancer progression [24]. Kyoto encyclopedia of genes and genomes (KEGG) enrichment analysis (Fig. 3D) revealed pathways impacted by the significant proteins, including systemic lupus erythematosus and neutrophil extracellular trap formation, both have been reported to promote colon cancer metastasis [25]. The molecular complex detection (MCODE) networks [26] showed protein–protein interactions gathered into 11 networks (Fig. 3E). The most complex network, MCODE1, represents platelet activation, signaling, aggregation, and degranulation. The second network is related to the initial triggering of complement activation and cascade. Another significant network, MCODE3, is composed of apolipoproteins and is associated with lipid-related processes.

**Fig. 4:**
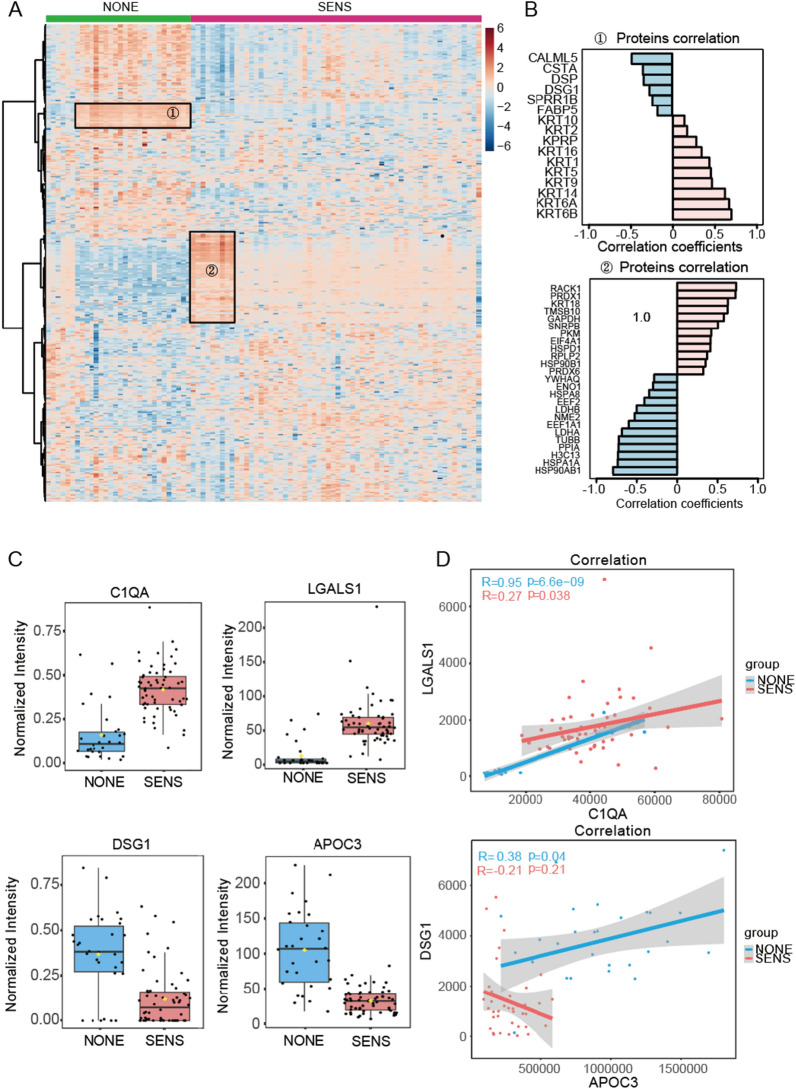
**A** Comparison Group SENS and group NONE with Benjamin-Hochberg FDR corrected t-test p value (< 0.05) passing proteins. The horizontal axis is all 90 samples analyzed in the study and vertical axis listed all quantified 804 proteins. Dendrogram for samples is shown on top of the heatmap, and the NONE group in green squares and the SENS group in red squares. The dark blue to dark red color gradient denotes lower to higher expression. **B** Protein correlation of two selected cluster from heatmap significant enrichment. Co-regulated proteins labeled the same color. Red and blue colored proteins present positive and negative correlated with group separation, respectively.**C** Boxplot of highly regulated protein expression, C1QA and LGALS1 for up-regulated; APOC3 and DSG1 for down-regulated. SENS group colored in red, and NONE group colored in blue. **D** Scatter plot between up regulated proteins C1QA and LGALS1, and Down regulated proteins APOC3 and DSG1, respectively. Dots are values of protein expression with a shadow of 95% confident interval

To explore patterns of protein expression across the patient cohorts, we performed hierarchical clustering of 804 quantified proteins filtered out from 831 proteins, in which proteins exhibited more than a 30% missing value rate across all samples were removed, visualized as a heatmap in Fig. 4A. Interestingly, this unsupervised analysis identified two significant clusters (circled and labeled), demonstrating distinct expression profiles in part of the NONE group and SENS group, respectively. We further examined the correlations between these clustered proteins and patient group classification by pattern search. Cluster 1 exhibited a diverse range of both positive and negative correlations with group classification. For instance, proteins like type I cytoskeletal 10 (KRT10) and type II cytoskeletal 2 epidermal (KRT2) displayed positive correlations, while fatty acid-binding protein (FABP5), cornifin-B (SPRR1B), and desmoglein-1 (DSG1) exhibited negative correlations with patient group classification. Similarly, cluster 2 revealed a variety of expression patterns within the proteins, indicating considerable heterogeneity within these groups. These findings underscore the complexity of the CRC-FOLFOX plasma proteome and emphasize the need for a comprehensive analysis of protein markers to distinguish patients with distinct clinical outcomes.

Furthermore, we observed significant differences in the expression levels of specific up- and down-regulated proteins between the SENS and NONE groups, spanning a wide range of intensities. These highly dysregulated proteins were found to be involved in various biological processes. For example, galectin-1 (LGALS1) was downregulated in the NONE group (Fig. 4C) and is known to play a role in regulating apoptosis, cell proliferation, and cell differentiation in carbohydrate metabolism. Previous studies have associated LGALS1 downregulation with poor prognosis in CRC [25]. On the other hand, apolipoprotein C-III and apolipoprotein A-II were significantly upregulated in the study (Fig. 4C), and are involved in maintaining blood function, potentially contributing to chemotherapy resistance. We further examined the correlation patterns of these highly regulated proteins. LGALS1 and complement C1q subcomponent subunit A (C1QA), both up-regulated proteins, exhibited a high correlation in the NONE group (R = 0.95) but a weaker correlation in the SENS group (R = 0.27) (Fig. 4D). Additionally, another up-regulated protein, protein disulfide-isomerase (P4HB), demonstrated a strong correlation with LGALS1 in both groups, with Pearson correlation coefficients of 0.91 and 0.83, respectively. In the case of downregulated proteins, apolipoprotein C-III (APOC3) and desmoglein-1 (DSG1) displayed a positive correlation in the NONE group (R = 0.38) but a negative correlation in the SENS group (R = -0.21). Both apolipoprotein A-II (APOA2) and apolipoprotein C-II (APOC2) were downregulated in both groups and exhibited similar correlations (Fig. 4D). These correlation patterns suggest that no single protein consistently changes in response to FOLFOX treatment in CRC patients. However, due to the lack of healthy individuals' samples and limited follow-up data, we were unable to directly assess the survival impact of these corresponding genes. To gain insights into the potential survival impact, we examined the disease-free survival (DFS) curve of these genes on gene expression profiling interactive analysis (GEPIA). The results, shown in Additional file 1: Fig. S1, indicated that low expression of C1QA and LGALS1 was associated with better patient survival, whereas high expression of P4HB was related to longer survival. It is important to note that individual gene expression patterns may not precisely align with the plasma protein expression profiles observed in our study. This discrepancy could be attributed to differences between tissue leakage proteins in plasma and solid tumors themselves. Additionally, proteins may be subject to multiple regulations in response to FOLFOX treatment, and individual gene expression alone may not solely impact DFS. Further investigations and validations are warranted to understand the potential survival impact and clinical significance of these proteins in CRC patients undergoing FOLFOX chemotherapy (Additional files 2, 4).

### Prognostic prediction of FOLFOX-treated CRC patients by machine learning

**Fig. 5:**
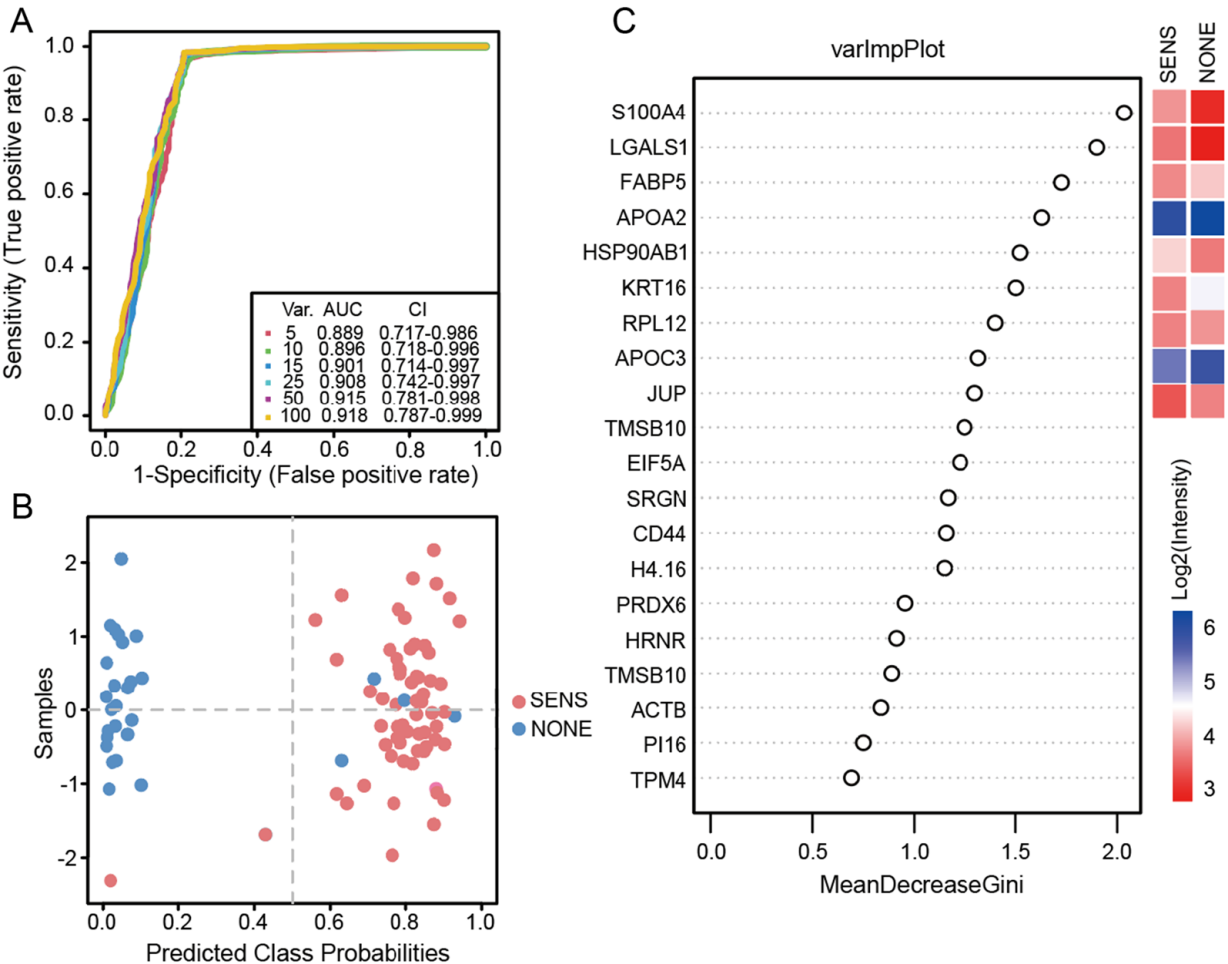
Machine Learning model. **A** ROC curve based on fivefold cross validation repeated 3 times. Each string represents a model with corresponding variables. **B** Classifier of predicted class probabilities for each sample. **C** Significant Features selected by Mean Decrease Gini index after 5-cross validation. 15 selected proteins are shown

In our study, we employed a hypothesis-free machine learning method called Random Forest to explore the possibility of predicting the curative effect of FOLFOX treatment on Stage II/III CRC patients. For this analysis, we utilized the 115 dysregulated proteins as signatures. The samples were randomly divided into two sets, with 40 SENS group and 20 NONE group samples used as the training set, and the remaining samples as the validation set. We generated multiple models with varying numbers of features (1 to 115) based on fivefold cross-validation (Fig. 5A). The generated models exhibited excellent performance, as evaluated using the receiver operating characteristic (ROC) curve. After thorough evaluation, we selected the model consisting of 25 preferential variables, which achieved an area under the ROC curve (AUC) of 0.908, with a 95% confidence interval of 0.742–0.997. This selected model demonstrated high accuracy, correctly classifying most of the patients into their respective groups. Only 4 SENS group and 2 NONE group patients were misclassified, resulting in over 93% accuracy (Fig. 5B). The top 20 protein signatures of this selected model are shown in Fig. 5C. Among these signatures, protein S100 calcium-binding protein A4 (S100A4) emerged as the most important variable, and it has been previously reported as a prognostic biomarker for colorectal cancer [27]. Another important signature, LGALS1, is known to undergo significant changes during colorectal cancer development and metastasis, and it has been implicated in various normal and pathological processes [25, 28]. Fatty acid-binding protein 5 (FABP5), a fatty acid-binding protein, was also identified as a crucial signature in the model and has been recognized as a novel target for its regulatory role in lipid metabolism in colorectal cancer [29]. Furthermore, a panel of 9 proteins was selected based on their high Gini index (higher than 1.3). This panel included highly up-regulated proteins such as LGALS1, S100A4, large ribosomal subunit protein uL11 (RPL12), and heat shock protein HSP 90-beta (HSP90AB1), highly down-regulated proteins like FABP5 and type I cytoskeletal 16 (KRT16), and slightly down-regulated proteins APOA2, APOC3, and junction plakoglobin (JUP). This combination of biomarker panels holds significant potential as a powerful prediction model for assessing the curative effect of FOLFOX treatment in CRC patients. Overall, our machine learning approach using the plasma proteome data has demonstrated promising results for predicting treatment outcomes in CRC patients undergoing FOLFOX chemotherapy. However, further validation studies with larger patient cohorts are essential to establish the clinical utility and robustness of this prediction model.

### Parallel reaction monitoring (PRM) validation

**Fig. 6:**
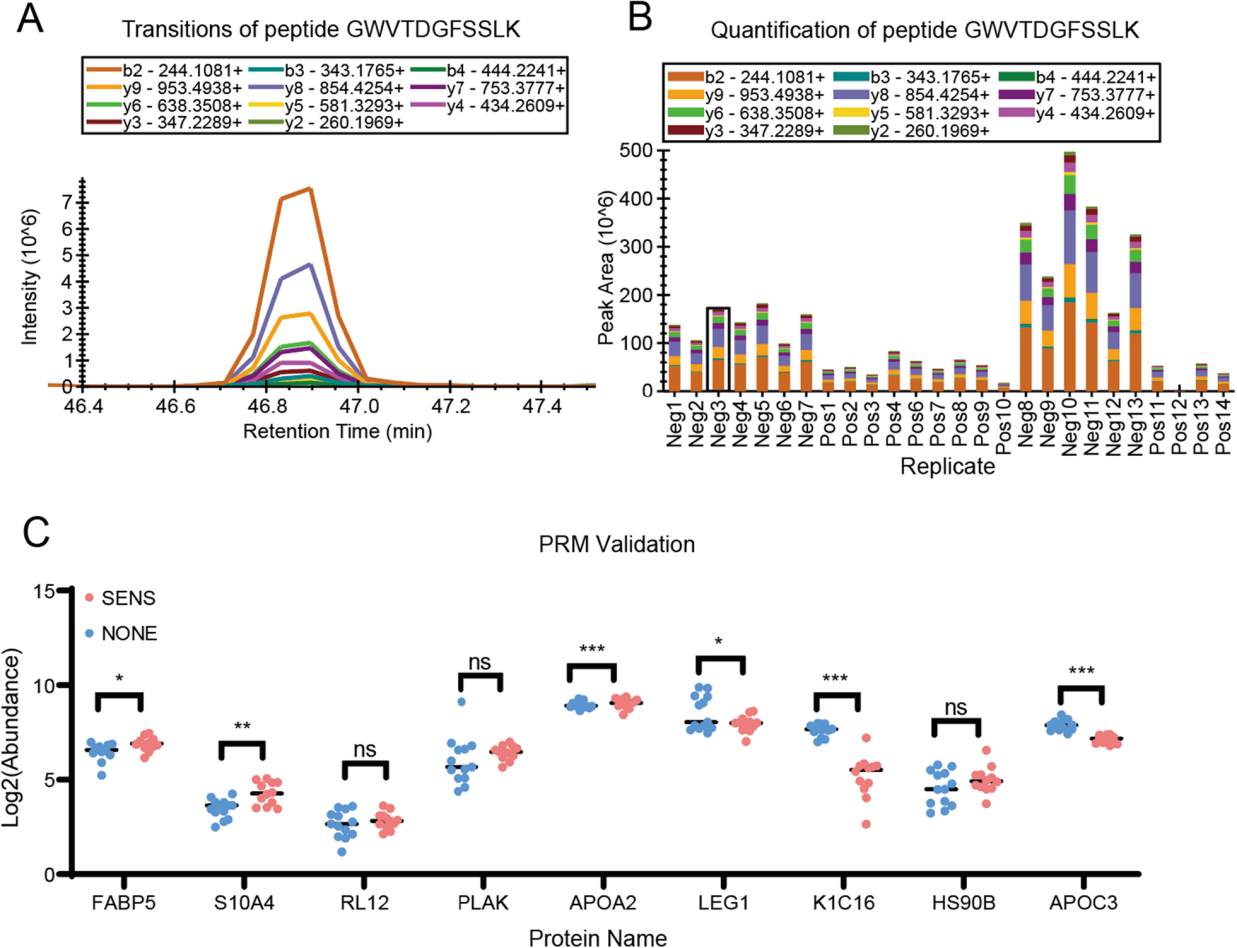
PRM validation. **A** Transitions of peptide GWVTDGFSSLK selected for quantification. **B** Quantification of peptide GWVTDGFSSLK. Each color presents a transition ion and corresponding bar graph presents peak area values. **C** Protein Abundance comparison across 26 validated samples including 13 SENS and 13 NONE group samples respectively

Parallel reaction monitoring (PRM) is a targeted mass spectrometry-based method that allows for precise and sensitive quantification of specific peptides or proteins in complex biological samples. In our study on predicting the curative effect of FOLFOX treatment on CRC patients, PRM validation is a crucial step in confirming the significance and reliability of the identified protein panel. To validate the findings from the discovery cohort, we collected a new cohort of 26 CRC patients, including 13 patients in the SENS group and 13 in the NONE group. We selected targeted peptides for the panel of 9 proteins identified in the discovery cohort. An example of the APOC3 peptide transition peak and quantification analysis is illustrated in Fig. 6A, B. By comparing the protein abundance in the two groups across these 9 proteins (Fig. 6C), we observed significant changes in 6 proteins. Notably, a panel of 5 proteins, namely S100A4, RL12, KRT16, HSP90AB1 and APOC3, exhibited expression changes consistent with the results obtained from the machine learning analysis, with 3 of these proteins showing statistical significance. The PRM validation results strengthen the robustness of our identified protein panel as potential biomarkers for predicting the curative effect of FOLFOX treatment in CRC patients. The concordance between the machine learning analysis and the PRM validation provides additional evidence for the reliability and accuracy of our prediction model. In conclusion, the use of PRM validation in our study further supports the potential clinical utility of the identified protein panel as a powerful tool for assessing treatment outcomes in CRC patients undergoing FOLFOX chemotherapy. However, further validation in larger patient cohorts and additional functional studies will be essential to fully establish the clinical value of these protein markers.

## Discussion

In this study, we utilized a plasma proteomics approach using the SISPROT-DIA workflow and LC–MS/MS technology to investigate post-diagnosis II/III stage CRC patients treated with FOLFOX chemotherapy. Profiling the plasma proteome allowed us to explore disease progression and identify potential diagnostic methods for new biomarkers to evaluate long-term treatment and predict treatment efficacy. By analyzing protein regulation in the SENS and NONE groups, we developed a protein panel that effectively classified and predicted the outcomes of FOLFOX treatment. While some proteins in the panel were validated, further validation using additional methods, such as isotopic labeling peptides for absolute quantification or ELISA with a larger cohort of patients, is needed to enhance the robustness and reliability of the panel. Considering the longitudinal changes in the protein panel as patients undergo long-term FOLFOX chemotherapy is essential. Monitoring the dynamic changes in the panel over time could provide valuable insights into treatment effectiveness and guide potential treatment adjustments. Collecting samples at different time points during treatment would offer a more comprehensive understanding of treatment responses. Moreover, to improve the clinical relevance of our findings, collecting more clinical information beyond traditional tumor markers (CEA, CA19-9, and CA125) is important. For example, incorporating the neutrophil-albumin ratio (NAR) as a prognostic signature for CRC patients after surgery could provide additional valuable data. Including more detailed clinical information and patient classification would contribute to a more comprehensive analysis. For instance, location of CRC tumor could induce bias in plasma proteome [30]. Integration of genomics and metabolomics data with proteomics through multi-omics approaches [31, 32] could provide more accurate insights. The use of advanced artificial intelligence and machine learning algorithms with these multidimensional datasets could enhance biomarker discovery and predictive modeling. Increasing the sample size, applying appropriate statistical analyses, and validating findings through multiple independent approaches are crucial to ensuring robust and reliable conclusions. Despite its limitations, our MS-based proteomics workflow demonstrates its feasibility for biomarker discovery. Our study emphasizes the essential role of proteomics in identifying potential biomarkers for predicting the response to FOLFOX treatment in CRC patients. The integration of sophisticated LC–MS/MS and machine learning methodologies has the potential for developing a robust protein panel. Our ultimate goal is to translate these proteomic insights into a practical and reliable predictive tool. Such a tool could significantly empower oncologists to accurately prognosticate the efficacy of FOLFOX treatment on an individualized basis, thereby enhancing treatment decision-making and optimizing therapeutic outcomes for CRC patients.

### Supplementary Information

**Additional file 1: Figure S1.** Disease free survival of C1QA, LGALS1 and P4HB.

**Additional file 2: Table S1. **Characteristics of recruited patients.

**Additional file 3: Table S2.** Original quantitative data matrix.

**Additional file 4: Table S3.** Pathway enrichment.

## Data Availability

The mass spectrometry proteomics data have been deposited to the ProteomeXchange Consortium via the PRIDE partner repository with the dataset identifier PXD044201. Reviewer account details: Username: reviewer_pxd044201@ebi.ac.uk Password: 3qlmcN0G.
