# Supplementary figures and images for: Plasma proteomic characterization of colorectal cancer patients with FOLFOX chemotherapy by integrated proteomics technology
Source: Clin Proteomics. 2024 Apr 5;21:27. doi: 10.1186/s12014-024-09454-z (PMC10998366; doi:10.1186/s12014-024-09454-z)

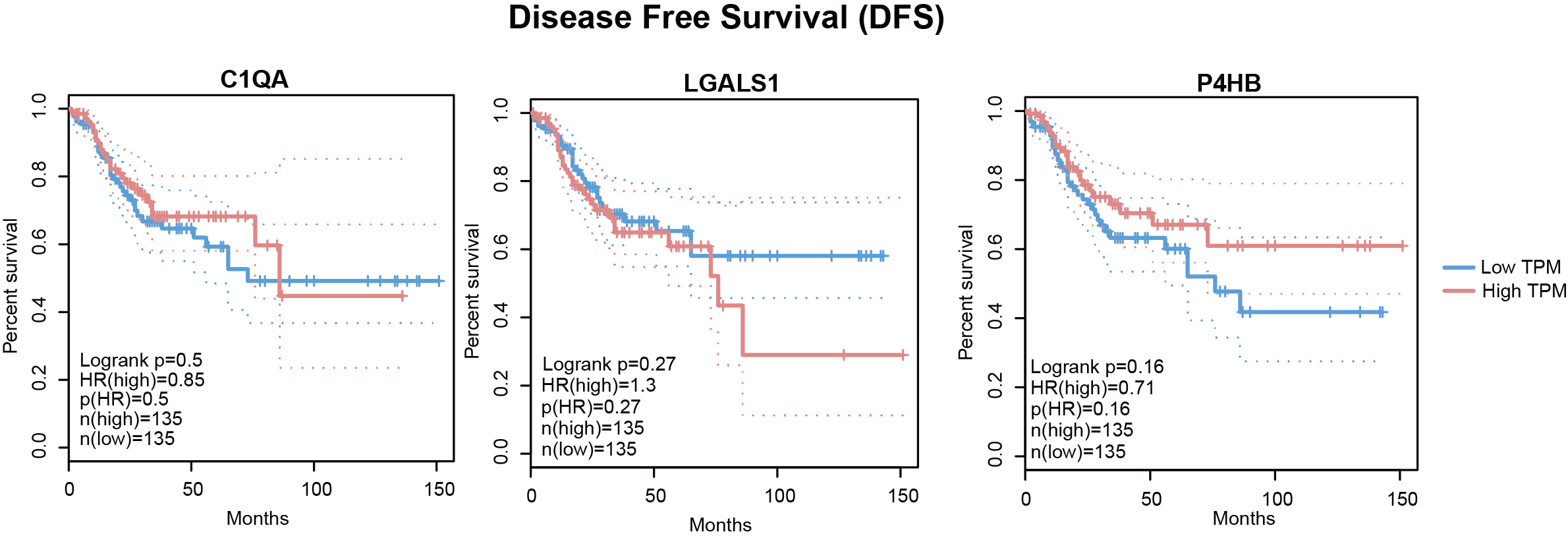

Supplement: Supplementary file 1 — Additional file 1: Figure S1. Disease free survival of C1QA, LGALS1 and P4HB. [file 12014_2024_9454_MOESM1_ESM.tif]
